# Sialic acid–modified der p 2 allergen exerts immunomodulatory effects on human PBMCs

**DOI:** 10.1016/j.jacig.2023.100193

**Published:** 2023-11-20

**Authors:** Brigitte-Carole Keumatio Doungtsop, Eleonora Nardini, Hakan Kalay, Serge A. Versteeg, Joyce Lübbers, Gaby van Barneveld, Eveline R.J. Li, Sandra J. van Vliet, Ronald van Ree, Esther C. de Jong, Yvette van Kooyk

**Affiliations:** a Department of Molecular Cell Biology and Immunology, Amsterdam UMC location Vrije Universiteit Amsterdam, Amsterdam, The Netherlands; b Inflammatory Diseases Research Program, Amsterdam Institute for Infection and Immunity, Amsterdam, The Netherlands; c DC4U Technologies, Abcoude, The Netherlands; d Department of Experimental Immunology, Amsterdam UMC location Amsterdam Medical Center, Amsterdam, The Netherlands

**Keywords:** HDM, Der p 2, antigen-presenting cells, CD4^+^ T cells, IL-5, AIT, allergic asthma, sialic acids, Siglecs

## Abstract

**Background:**

House dust mite extract–based allergen immunotherapy (AIT) to treat house dust mite allergy is substantially effective but still presents some safety and efficacy concerns that warrant improvement. Several major allergen-based approaches to increase safety and efficacy of AIT have been proposed. One of them is the use of the group 2 allergen, Der p 2.

**Objective:**

We sought to investigate the immunomodulatory effects of sialic acid–modified major allergen recombinant Der p 2 (sia-rDer p 2) on PBMCs from healthy volunteers.

**Methods:**

We activated PBMCs with anti-CD3/CD28 antibodies and incubated them at 37°C for 6 days in the presence or absence of either native rDer p 2 or α2-3 sialic acid–modified rDer p 2 (sia-rDer p 2). We assessed the changes in CD4^+^ T-cell activation and proliferation by flow cytometry and changes in T-lymphocyte cytokine production in cell culture supernatant by ELISA.

**Results:**

We observed that PBMCs treated with sia-rDer p 2 presented with a markedly decreased expression of CD69 and an increased abundance of LAG-3^+^ lymphocytes compared with cells treated with rDer p 2. Moreover, PBMCs treated with sia-rDer p 2 showed a reduced production of IL-4, IL-13, and IL-5 and displayed a higher IL-10/IL-5 ratio compared with rDer p 2–treated PBMCs.

**Conclusions:**

We demonstrate that sia-rDer p 2 might be a safer option than native rDer p 2 for Der p 2–specific AIT. This is most relevant in the early phase of AIT that is often characterized by heightened T_H_2 responses, because sia-rDer p 2 does not enhance the production of T_H_2 cytokines.

The standard therapeutic strategies for managing immune dysregulation, such as the excessive immune response in allergic reactions, mostly involve the use of immunosuppressive drugs, such as corticosteroids, Janus kinase, and calcineurin inhibitors, which lead to generalized immunosuppression.^1^ Because of the multiple side effects associated with immunosuppressive drugs, preventing or attenuating immune dysregulation in a specific manner is highly desirable. Most strategies under investigation for inducing targeted immune tolerance often use (nano- or lipid) particles encapsulating allergens and/or immunosuppressive compounds.^2^, ^3^, ^4^ These strategies generally aim to alter antigen-presenting cells (APCs) to suppress inflammatory T cells and induce regulatory T (Treg) cells.^5^

Directly targeting APCs including dendritic cells (DCs), monocytes, and B cells is an attractive strategy to modulate T-cell function and to induce antigen-specific tolerance.^5^ In particular, inhibitory receptors expressed by APCs, such as the Siglec (sialic acid–binding immunoglobulin-like lectin) family of carbohydrate-binding proteins, are a good target. Siglecs recognize sialic acid–containing glycoproteins and glycolipids and can be targeted to induce immunosuppressive responses.^5^^,^^6^ Most of the Siglecs, including Siglec-9 and Siglec-10, discussed in this study possess immunoreceptor tyrosine-based inhibitory motifs, which transmit immune deactivating signals on recognition of their ligand (sialic acids) and can thus be therapeutically exploited for the management of inflammatory diseases.^6^

We previously demonstrated that *ex vivo* and *in vivo* DC-targeting of α2-3 sialic acid–modified antigen to the mouse homolog of human Siglec-9 (ie, Siglec-E) drove naive CD4^+^ T-cell differentiation into antigen-specific Treg cells.^7^ Moreover, DCs treated with sialic acid–modified antigen dampened T-cell differentiation to effector T cells even in the presence of native antigen-loaded DCs.^7^ Also, exposing LPS-stimulated human monocyte–derived DCs to Siglec-9 ligands, α2-3 sialic acids, or anti–Siglec-9 antibodies suppressed the production of IL-6 and IL-12.^8^^,^^9^ Together, these reports show that targeting Siglec-9 inhibits T_H_1 responses. However, very little is known about whether Siglec-9–mediated interferences can also alter allergen-associated T_H_2 responses, characterized by the secretion of IL-4, IL-5, and IL-13.

We therefore evaluated the immunomodulatory effects of α2-3 sialic acid–modified antigen on the T_H_2 arm of T_H_ cells in human PBMCs using Der p 2 as a model antigen. Der p 2 is one of the established major allergens from house dust mite (HDM) and is an important risk factor for the development of allergic rhinitis and asthma.^10^^,^^11^ Consequently, research into the use of Der p 2 for allergen immunotherapy (AIT) against Der p 2–HDM allergy instead of HDM extracts has grown in the past decades because AIT with HDM still presents some safety and efficacy concerns that warrant improvement.^4^ Exploiting the sialic acid–Siglec axis by modifying Der p 2 via its 12 free lysine residues^12^ with sialic acids could provide Siglec-mediated crosslinking and signaling to attenuate allergen-specific T_H_2-mediated immune responses.^13^

## Methods

### α2-3 Sialic acid–recombinant Der p 2 binding to Siglec–human Fc chimeras

NUNC MaxiSorp plates (Greiner Bio-One, Fredensborg, Denmark) were coated with 10 μg/mL of recombinant Der p 2 (rDer p 2) or α2-3 sialic acid–rDer p 2 (sia-rDer p 2) overnight at room temperature. After washing with HBSS (Gibco, New York, NY), wells were blocked with carbo-free blocking buffer—glycoprotein-free blocking agent (Vector Laboratories, Newark, Calif) diluted 1:10 in HBSS for 1 hour at room temperature and subsequently incubated with Siglec-Fc chimeras and goat anti–human Fc-peroxidase antibody (Jackson Laboratory, Bar Harbor, Me). The following Siglec-Fc chimeras (R&D Systems, Minneapolis, Minn) were used: Siglec-2–hFc (1968-SL-050), Siglec-3–hFc (1137-SL-050), Siglec-7–hFc (1138-SL-050), Siglec-9–hFc (1139-SL-050), and Siglec-10–hFc (2130-SL-050).

For all colorimetric assays, plates were developed using 3,3′,5,5′-tetramethylbenzidine (Merck, Germany) as a substrate and H_2_SO_4_ as the stop solution. The iMark microplate absorbance reader (Bio-Rad) was used to measure absorbance at 450 nm.

### Cell Isolation and culture

Blood from healthy volunteers for the isolation of PBMCs was supplied by the Sanquin Blood Bank (Sanquin, Amsterdam, The Netherlands). Healthy donors (nonallergic) gave their written consent for the use of blood donation for research purposes. We performed an ImmunoCAP assay to confirm the allergic status of donors. PBMCs were isolated from whole blood by density gradient centrifugation (at 2000 rpm for 30 minutes) using Lymphoprep (Serumwerk, Serumwerk, Germany). After isolation, PBMCs were frozen and stored at −80°C (or in liquid nitrogen) until use. PBMCs were thawed and plated in 96-well round-bottomed plates (Greiner Bio-One, Alphen aan den Rijn, The Netherlands) at a density of 10  ×  10^6^ cells/mL in RPMI 1640 (Thermo Fisher, Waltham, Mass) supplemented with 10% inactivated FBS (Biowhittaker, Switzerland), 1% glutamine (Thermo Fisher), and 1% penicillin-streptomycin (Lonza, Basel, Switzerland). The PBMCs were left inactivated or activated with plate-bound anti-CD3 (10 μg/mL) (clone SPV-T3b, Monoclonal Antibody Facility Department of Molecular Cell Biology and Immunology, Amsterdam, The Netherlands) and soluble anti-CD28 (2 μg/mL) (Sanquin). The activated PBMCs were subsequently either left untreated (control) or treated with rDer p 2 (10 μg/mL) or sia-rDer p 2 (10 μg/mL) for 6 days at 37°C and 5% CO_2_ in humidified air. Supernatants were harvested and stored at −20°C for cytokine measurements and cells were harvested for flow cytometry.

### Prevention or reversion of PBMC responses

To investigate whether sia-rDer p 2 can revert an existing inflammation involving rDer p 2, anti–CD3-/CD28-activated PBMCs (10 ×  10^6^ cells/mL) were first treated with rDer p 2 (10 μg/mL) for 2 days, after which cells were washed with RPMI medium and sia-rDer p 2 (10 μg/mL) was added to the cells and cultured for another 4 days. To investigate whether sia-rDer p 2 can prevent inflammation involving rDer p 2, anti–CD3-/CD28-activated PBMCs were first treated with sia-rDer p 2 (10 μg/mL) for 2 days, cells were washed with RPMI medium, and rDer p 2 (10 μg/mL) was added to the cells and cultured for another 4 days. This setup was adapted from that previously described.^14^

### Flow cytometry

For the characterization of the different cell populations within PBMCs and the evaluation of Siglec expression, cells (1 × 10^6^/well) were stained with mAbs (Table I) for 30 minutes on ice in a 96-well V-bottomed plate (Greiner Bio-One). Antibodies were diluted in PBS containing 0.1% BSA (Roche, Rotkreuz, Switzerland), 0.02% sodium azide, Fc block (in-house), and True-Stain monocyte blocker (BioLegend, Amsterdam, The Netherlands). Fixable Viability stain, Zombie NIR (BioLegend), or LIVE DEAD Blue (Thermo Fisher) was used to stain dead cells for 15 minutes before staining surface markers. For intracellular Ki67, IL-10, and FoxP3 detection, cells were incubated with 0.5 μg/mL of phorbol 12-myristate 13-acetate (Sigma-Aldrich, Sofia, Bulgaria) and 1 μg/mL of ionomycin (Sigma-Aldrich) in the presence of GolgiStop and Golgiplug (BD Biosciences, Erembodegem, Belgium) for 4 hours. After staining cell surface markers, cells were fixed-permeabilized using FoxP3/Transcription Factor Staining Buffer Set (Thermo Fisher, Waltham, Mass) as directed by the manufacturer and stained with intracellular antibodies (Table I). Labeled cells were fixed with 1% paraformaldehyde (Electron Microscopy Sciences, Hatfield, Pa) and stored at 4°C until acquisition. Fluorescence minus 1 controls were prepared for each cell marker and used for gating. Stained samples were acquired with the 4- or 5-laser Aurora flow cytometer (Cytek Biosciences, Amsterdam, The Netherlands). FCS files were analyzed using FlowJo software v10.8 (BD Biosciences).

**Table I:** mAbs used for flow cytometry, including clone, manufacturer, and catalog number

Fluorochrome	Antigen	Clone	Manufacturer	Catalog no.
PE-Cy5	CD33	WM53	BioLegend	303406
Atto647	CD56	NBL-1	Monoclonal Antibody Facility MCBI	NA
PE-CF-594	CD56	NCAM16.2	BD Horizon	564849
BV510	CD3	UCHT1	BioLegend	300448
APC-Cy7	CD3	UCHT1	BioLegend	300425
BV605	CD3	UCHT1	BioLegend	300460
PerCP	CD4	OKT4	BioLegend	317432
BV711	CD4	RPA-T4	BioLegend	300558
BV421	CD8	RPA-T8	BioLegend	301036
BV750	CD19	HIB19	BioLegend	302262
BUV496	CD19	SJ25C1	BD Biosciences	612938
FITC	CD69	FN50	BD Biosciences	555530
BUV737	CD25	2A3	BD Biosciences	612806
PE-Dazzle594	CRTH2	BM16	BioLegend	350126
CXCR3	PE-Fire810	G025H7	BioLegend	353760
BV605	CD49b	AK-7	BD Biosciences	742646
APC-Cy7	LAG-3	7H2C65	BioLegend	369219
Alexa Fluor 700	PD-1	EH12.2H7	BioLegend	329952
V450	FoxP3	259D/C7	BD Biosciences	560459
APC	Ki67	Ki-67	BioLegend	350513
PE-Cy7	IL-10	JES-9D7	BioLegend	501420
eFlour450	CD14	61D3	Invitrogen	48-0149-42
BV605	CD14	M5E2	BioLegend	301834
BV480	HLA-DR	G46-6	BD Biosciences	566113
BUV563	HLA-DR	L203.rMAb	BD Biosciences	752494
BV510	CD16	3G8	BioLegend	302048
PE-Cy5	CD16	3G8	BioLegend	302010
BV785	CD123	6H6	BioLegend	306032
BV650	CD11c	3.9	BioLegend	301638
FITC	CD1c	L161	SONY Biotechnologies	2257585
BV711	CD141	1A4	BD Biosciences	563155
PE-Cy7	Siglec-1	7-239	BioLegend	346014
PE	Siglec-2	HIB22	BioLegend	302506
BV650	Siglec-3	P67.6	BioLegend	366612
BB515	Siglec-5	194128	BD Biosciences	565198
Alexa Fluor 700	Siglec-6	767389	R&D Systems	FAB2859N
PerCP vio700	Siglec-7	REA214	Miltenyi Biotec	130-100-979
Alexa Fluor 488	Siglec-9	191240	R&D Systems	FAB1139G
APC	Siglec-10	5G6	BioLegend	347606

*FITC*, Fluorescein isothiocyanate; *MCBI,* Molecular Cell Biology and Immunology; *NA*, not applicable; *PE*, phycoerythrin.

### Binding of sia-rDer p 2 to PBMCs

PBMCs (1 × 10^6^/well) were washed with 0.5% BSA in HBSS (ice-cold) in a 96-well V-bottomed plate. rDer p 2 or sia-rDer p 2 (10 μg/mL) or 10 μg/mL polyacrylamide–α2-3 biotin (positive control) was added to the cells and incubated for 1 hour at 37°C. Subsequently, 10 μg/mL of biotinylated anti–Der p 2 antibody (Absolute Antibody, Amsterdam, The Netherlands) was added and incubated for 45 minutes at 4°C. Cells were then stained with mAbs to characterize PBMC subsets (Table I) and streptavidin-phycoerythrin to detect binding (Jackson Laboratories) for 30 minutes at 4°C. Stained cells were fixed in 1% paraformaldehyde and acquired with the 5-laser Aurora flow cytometer (Cytek Biosciences).

### Cytokine quantification

The concentrations of IL-4, IL-5, IL-10, IL-13, IL-17, and IFN-γ in supernatants were quantified by sandwich ELISA using the following anticytokine antibody pairs: IL-4 (Biosource; AHC0642 and AHC0749), IL-5 (BioLegend; 500902 and 501002), IL-10 (eBioscience; 14-7108-85 and 13-7109-85), IL-13 (Invitrogen; P130E and M130B), IL-17 (eBioscience; 14-7178-85 and 13-7179-85), and IFN-γ (eBioscience; 14-7318-85 and 13-7319-85). The capture antibody was coated in NUNC MaxiSorp plates (Greiner Bio-One) overnight at 4°C in 0.05 mol Na_2_CO_3_ buffer (pH 9.7) (Merck, Boston, Mass). The plates were blocked with 1% BSA for 1 hour at 37°C. Supernatants and biotinylated antibodies were added and plates were incubated for 2 hours at room temperature.

### Statistical analysis

Data are presented as mean ± SD (as indicated in figure legends). *P* values were determined by the Wilcoxon paired test using GraphPad Prism version 9 (GraphPad, San Diego, Calif) (as indicated in figure legends). Differences in values were considered significant at a *P* value of less than .05.

## Results

### Sia-rDer p 2 binds to APCs

**Fig 1:**
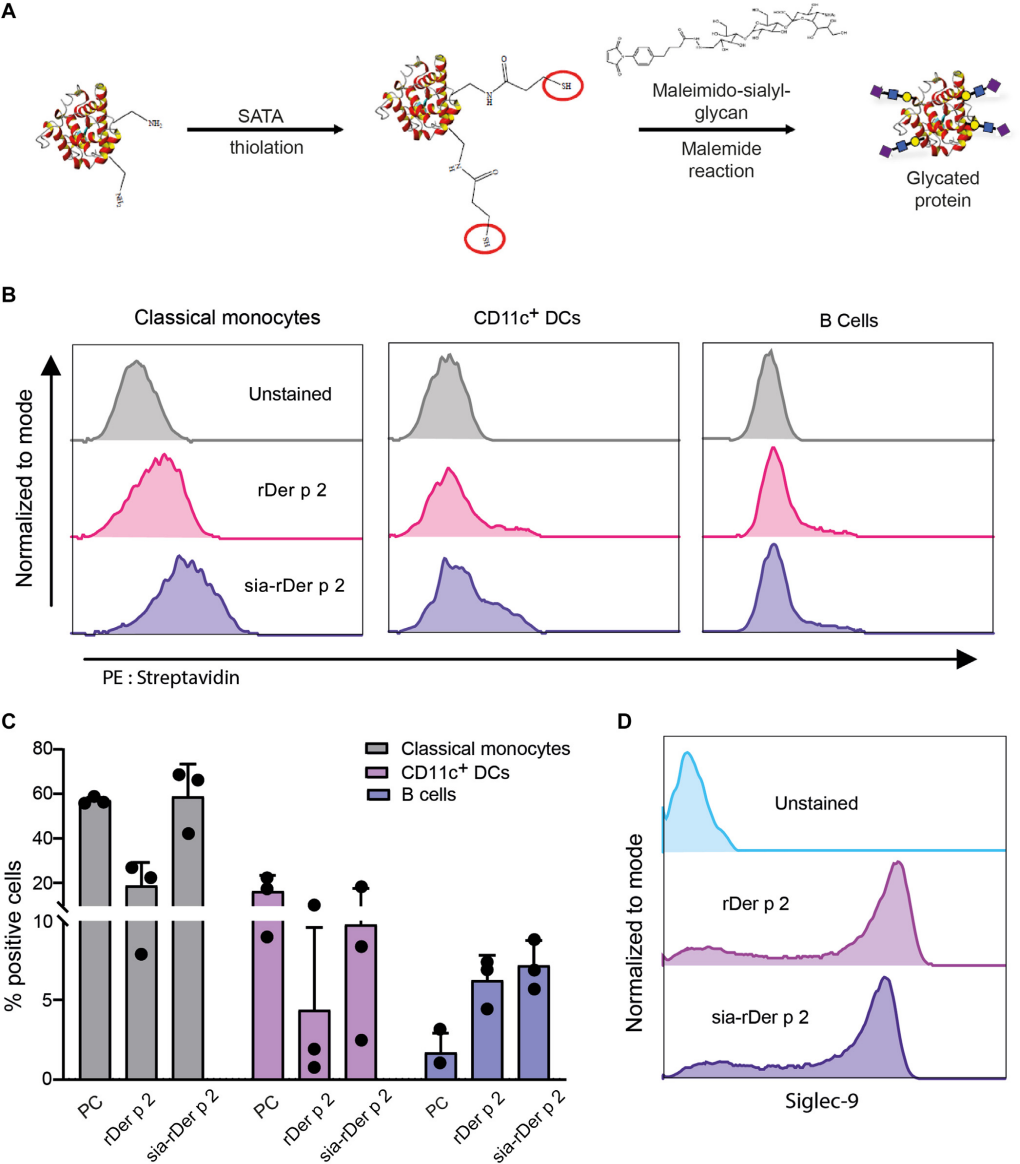
sia-rDer p 2 binds to Siglec-9 and/or Siglec-10 expressed on APCs. **A**, Schematic representation of the thiol-malemide reaction for the conjugation of α2-3 sialic acids to rDer p 2. **B**, Representative histograms showing the binding of rDer p 2 and sia-rDer p 2 on classical monocytes (CD14^+^CD16^+^), CD11c^+^ DCs, and B cells. **C**, The percentage of cells among monocytes, CD11c^+^ DCs, and B cells that bound polyacrylamide-α2-3 (positive control [PC]), rDer p 2, and sia-rDer p 2 (n = 3). Data are shown as mean ± SD. **D**, Representative histograms showing the expression of Siglec-9 on classical monocytes after incubation of cells with either rDer p 2 or sia-rDer p 2.

α2-3 sialic acids were chemically conjugated to rDer p 2 through a maleimide-thiol reaction to produce glyco-allergen conjugates (sia-rDer p 2) (see the Methods section in this article’s Online Repository at www.jaci-global.org) (Fig 1, *A*). We performed a lectin-binding ELISA to confirm the presence of α2-3 sialic acids on sia-Der p 2 (see the Methods section and Fig E1 in this article’s Online Repository at www.jaci-global.org). We then assessed whether sia-rDer p 2 would bind to selected Siglec-Fc chimeras and found that sia-rDer p 2 but not rDer p 2 interacted primarily with Siglec-9–Fc (Fig E1). To investigate whether sia-rDer p 2 would bind to Siglecs expressed on immune cells, we first measured the expression of Siglec-9 and Siglec-10 on the different immune cells within PBMCs (gating strategy; Fig E1). Monocytes and DCs but not B cells expressed high levels of Siglec-9, whereas CD14^−^CD16^+^ nonclassical monocytes, DCs, and B cells expressed Siglec-10 (Fig E1). CD4^+^ T cells did not express Siglec-9 or Siglec-10 (Fig E1). We demonstrate that sia-rDer p 2 bound to monocytes, DCs, and B cells (Fig 1, *B* and *C*), but not to CD4^+^ T cells (Fig E1). Binding of sia-rDer p 2 to these cells partially reduced the Siglec-9 expression on these cells on preincubation with sia-rDer p 2 (Fig 1, *D*), which may indicate partial receptor occupancy. Overall, we illustrate that sia-rDer p 2 bound to APCs and that this binding may be Siglec-mediated.

### T_H_1- and T_H_2-cell activation is equally suppressed by sia-rDer p 2

**Fig 2:**
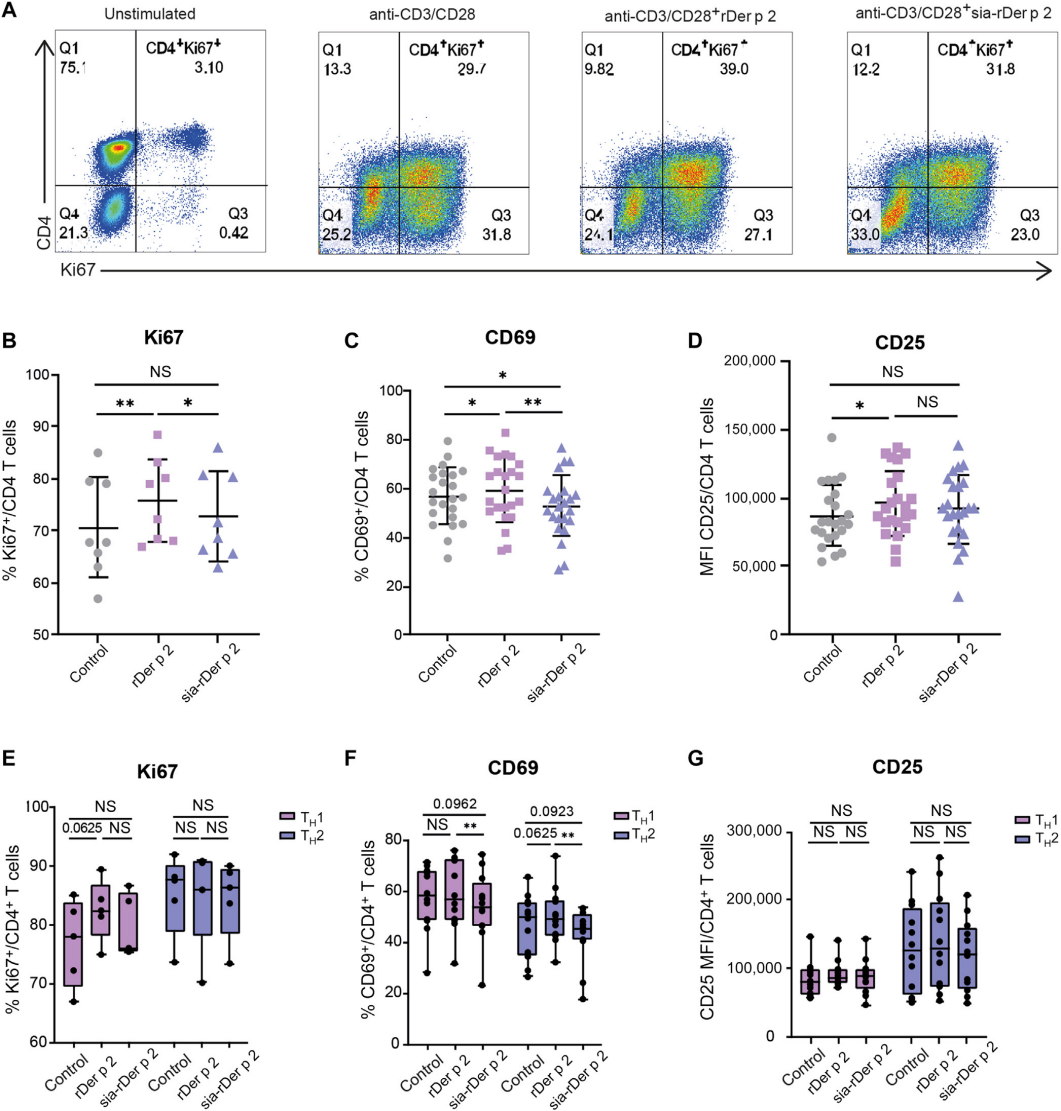
CD4^+^ T-cell activation is moderately suppressed by sia-rDer p 2. **A**, Representative dot plot showing the percentage of Ki67^+^CD4^+^ T cells, indicative of the percentage of proliferating CD4^+^ T cells. **B**, The percentage of Ki67^+^CD4^+^ T cells (n = 8). **C**, The percentage of CD69^+^CD4^+^ T cells (n = 23). **D**, The MFI of CD25 on CD4^+^ T cells (n = 23). **E**, The percentage of Ki67^+^ cells among CD4^+^CXCR3^+^ T_H_1 cells (*pink*) and CD4^+^CRTH2^+^ T_H_2 cells (*purple*) (n = 5). **F**, The percentage of CD69^+^ cells among CD4^+^CXCR3^+^ T_H_1 cells (*pink*) and CD4^+^CRTH2^+^ T_H_2 cells (*purple*) (n = 12). **G**, The MFI of CD25 on CD4^+^CXCR3^+^, T_H_1 cells (*pink*) and CD4^+^CRTH2^+^ T_H_2 cells (*purple*) (n = 12). All results are shown as mean ± SD. *MFI*, Mean fluorescence intensity; *NS*, not significant. ∗*P* < .05; ∗∗*P* < .01. Wilcoxon matched-pairs test.

To assess whether coincubation of anti–CD3-/CD28-activated PBMCs with sia-rDer p 2 altered the proliferation (expression of intracellular Ki67) and activation (surface expression of CD69 and CD25) of CD4^+^ T cells, PBMCs were cultured for 6 days with rDer p 2 or sia-rDer p 2 and assessed by flow cytometry (Fig 2; see also gating strategy in Fig E2 in this article’s Online Repository at www.jaci-global.org). Treatment of PBMCs with rDer p 2 significantly enhanced CD4^+^ T-cell proliferation and the expression of CD25 and CD69 compared with the control (baseline) (Fig 2, *A-D*), whereas sia-rDer p 2 did not. Of note, sia-rDer p 2 reduced the percentage of CD69^+^CD4^+^ T cells (Fig 2, *C*, and Fig E2, *B*) and the level of expression of CD69 (Fig E2) to lower than baseline levels. This reduction was similar in both T_H_1 lymphocytes (on the basis of expression of CXCR3) and T_H_2 lymphocytes (on the basis of CRTH2 expression) (Fig 2, *F*). The difference in proliferation and CD25 expression between the control, rDer p 2, and sia-rDer p 2 within T_H_1 and T_H_2 lymphocytes did not reach statistical significance (Fig 2, *E* and *G*). In conclusion, these data demonstrate that rDer p 2 enhances CD4^+^ T-cell activation and proliferation, whereas sia-rDer p 2 appears to suppress both T_H_1- and T_H_2-cell activation.

### sia-rDer p 2 modulates T_H_2 cytokine production but not T_H_1 or T_H_17 cytokines

**Fig 3:**
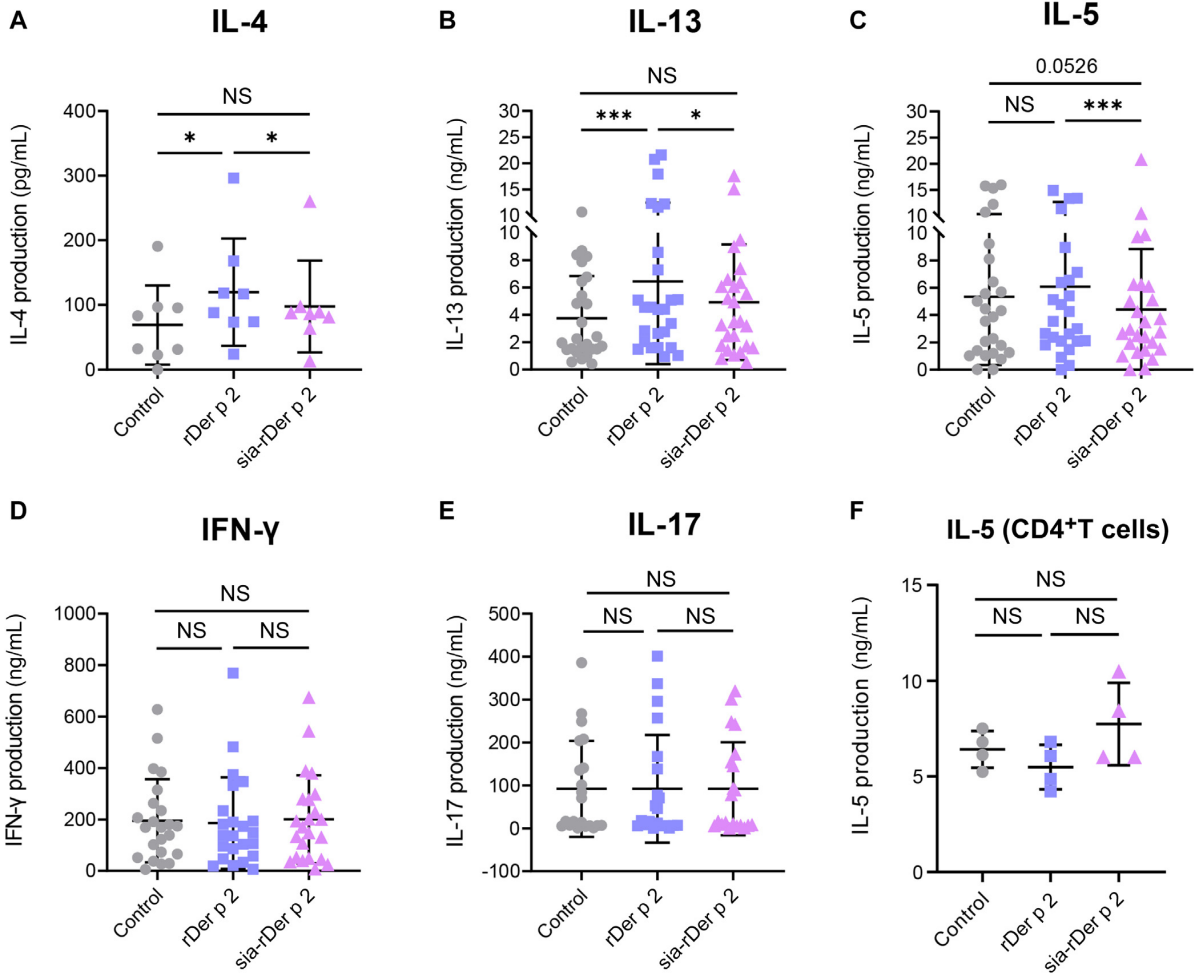
**A-E,** sia-rDer p 2 modulates the secretion of T_H_2 cytokines, but not T_H_1 or T_H_17 cytokines. Levels of IL-4 (n = 8) (Fig 3, *A*), IL-13 (n = 25) (Fig 3, *B*), IL-5 (n = 25) (Fig 3, *C*), IFN-γ (n = 12) (Fig 3, *D*), and IL-17 (n = 12) (Fig 3, *E*) in day 2 (IL-4) or day 6 cell culture supernatants were determined by ELISA. **F**, IL-5 was measured in day 6 cell culture supernatants after culturing anti–CD3-/CD28-activated pure CD4^+^ T cells with rDer p 2 or sia-rDer p 2. All results are shown as mean ± SD. *NS*, Not significant (Wilcoxon matched-pairs test). ∗*P* < .05; ∗∗∗*P* < .001.

Given that sia-rDer p 2 equally modulated both T_H_1- and T_H_2-cell activation, we investigated whether a similar effect would occur in their cytokine profiles. When compared with baseline, rDer p 2 upregulated the production of IL-4 (Fig 3, *A*) and IL-13 (Fig 3, *B*) but not IL-5 (Fig 3, *C*), IFN-γ (Fig 3, *D*), or IL-17 (Fig 3, *E*). However, sia-rDer p 2 downregulated the production of IL-5 (Fig 3, *C*) but did not affect IL-13, IL-4, IL-7, and IFN-γ (Fig 3, *A*, *B*, *D*, and *E*). Moreover, compared with rDer p 2, sia-rDer p 2 induced lower production of IL-13 and IL-4 (Fig 3, *A* and *B*). We then investigated whether the effects of sia-rDer p 2 on IL-5 were a result of direct binding to CD4^+^ T cells or mediated by APCs present in PBMCs by coincubating rDer p 2 or sia-rDer p 2 with pure CD4^+^ T cells activated with anti-CD3/CD28. The production of IL-5 was not affected (Fig 3, *F*). We can therefore conclude that sia-rDer p 2downregulates IL-5 secretion and that this may be via APC–T-cell interactions.

### The Treg/T_H_2 cytokine ratio is higher with sia-rDer p 2 than with native rDer p 2

**Fig 4:**
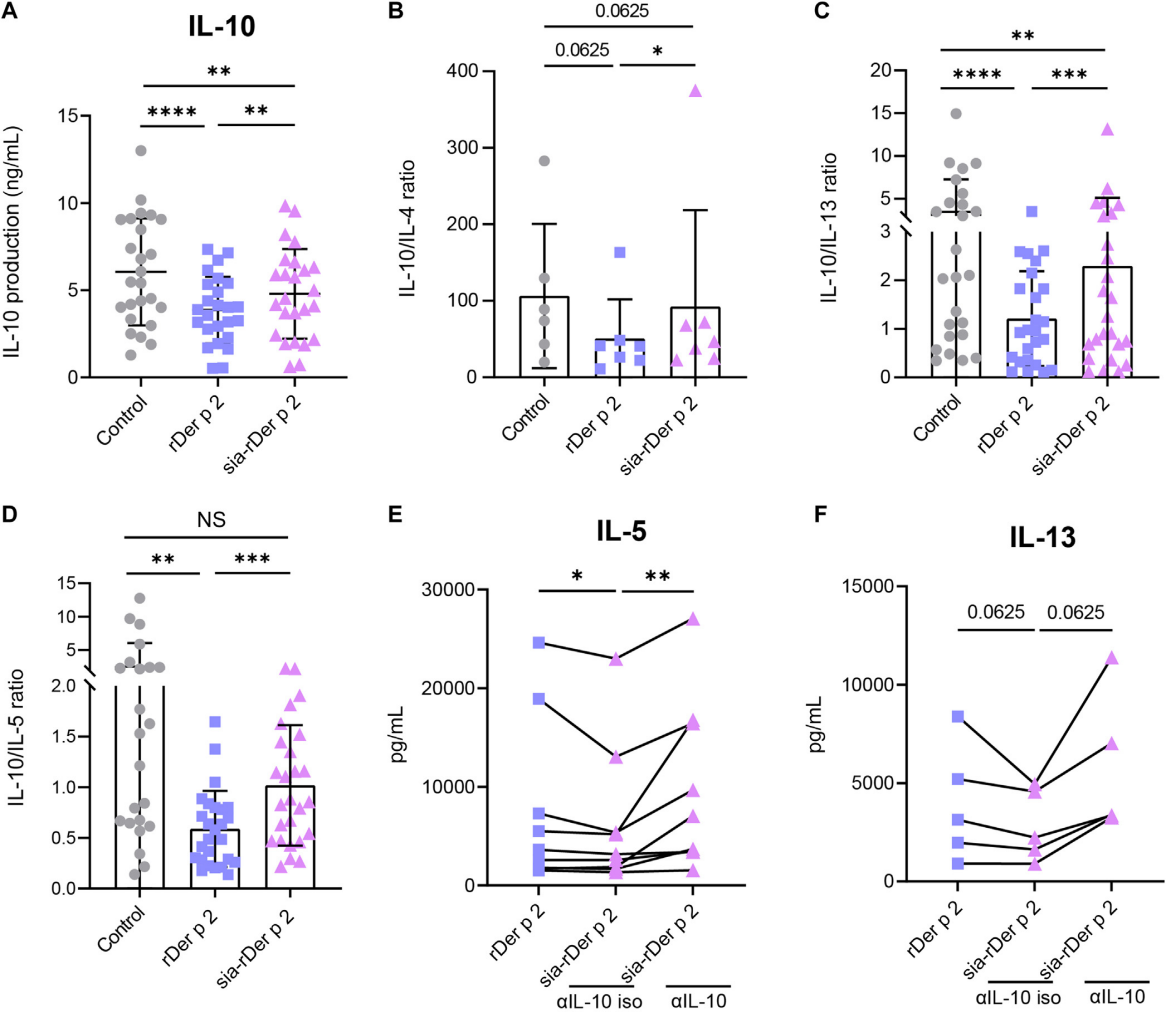
**A-D,** The Treg/T_H_2 ratio is higher with sia-rDer p 2 than with rDer p 2. The levels of IL-10 (n = 25) (Fig 4, *A*) were determined by ELISA in day 6 PBMC culture supernatants. The ratios of IL-10 to IL-4 (n = 8) (Fig 4, *B*), IL-10 to IL-13 (n = 25) (Fig 4, *C*), and IL-10 to IL-5 (n = 25) (Fig 4, *D*) were determined by dividing the values of IL-10 by the values of IL-4, IL-13, and IL-5, respectively, for each condition (control, rDer p 2 and sia-rDer p 2). PBMCs were activated with anti-CD3/CD28 antibodies and coincubated with either rDer p 2 or sia-rDer p 2 in the presence of anti–IL-10 (αIL-10) or an isotype antibody (αIL-10 iso) and cultured for 6 days. **E**, The levels of IL-5 produced when PBMCs were coincubated with rDer p 2 without anti–IL-10 or with sia-rDer p 2 and αIL-10 iso or anti–IL-1 αIL-10 (n = 9). **F**, The levels of IL-13 produced when PBMCs were coincubated with rDer p 2 without anti–IL-10 or with sia-rDer p 2 and αIL-10 iso or anti–IL-1 αIL-10 (n = 5). Results are shown as mean ± SD. *NS*, Not significant (Wilcoxon matched-pairs test). ∗∗*P* < .01; ∗∗∗*P* < .001; ∗∗∗∗*P* < .0001.

Successful AIT can be characterized by a high IL-10/IL-5 ratio.^12^ We therefore analyzed culture supernatants of anti–CD3-/CD28-activated PBMCs to determine the impact of rDer p 2 or sia-rDer p 2 on IL-10 production. IL-10 secretion was lower in PBMCs treated with rDer p 2 compared with the control. Sia-rDer p 2 also attenuated IL-10 secretion but to a lesser extent than rDer p 2 (Fig 4, *A*). We then determined the Treg/T_H_2 cytokine balance by calculating the ratio of IL-10 to IL-4, IL-13, and IL-5, respectively. We observed that the IL-10/IL-4, IL-10/IL-13, and IL-10/IL-5 ratios were higher in sia-rDer p 2–treated PBMCs than in rDer p 2–treated PBMCs (Fig 4, *B-D*). To further determine the role of IL-10 in the reduced expression of T_H_2-cell cytokines, we stimulated PBMCs in the presence of an IL-10–blocking antibody and analyzed the IL-5 and IL-13 production. In the presence of an IL-10–blocking antibody, Sia-rDer p 2–treated PBMCs produced more IL-5 and IL-13 (Fig 4, *E* and *F*). These data suggest that sia-rDer p 2, unlike rDer p 2, prevents the disruption of the Treg/T_H_2 cytokine balance and that IL-10 plays a role in the downregulation of IL-5.

### The frequency of LAG-3^+^ lymphocytes is higher in sia-rDer p 2–treated PBMCs than in rDer p 2–treated PBMCs

**Fig 5:**
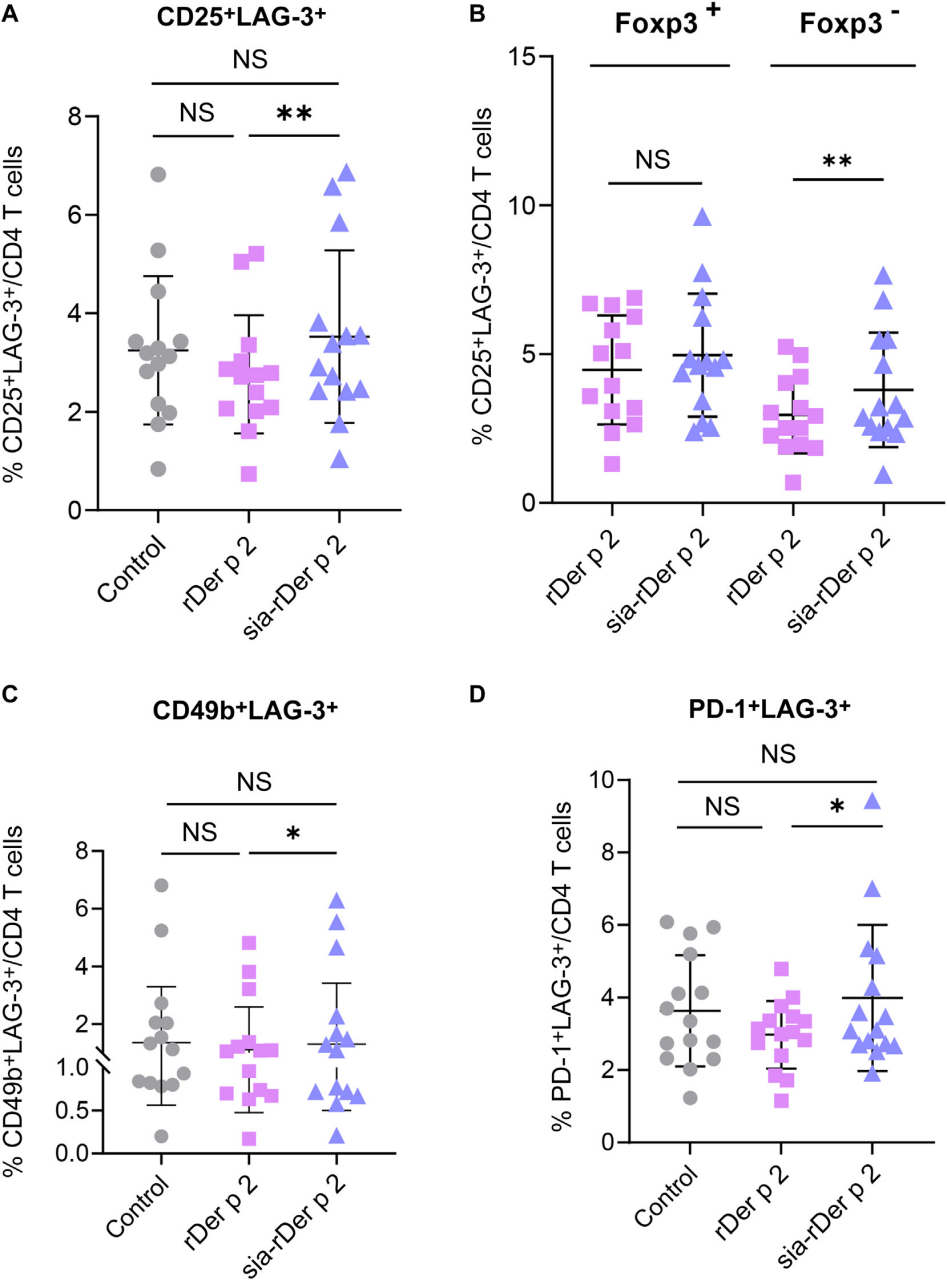
The frequency of LAG-3^+^ lymphocytes is higher in sia-rDer p 2–treated PBMCs. **A**, The percentage of CD25^+^LAG-3^+^CD4^+^ T cells in control, rDer p 2, or sia-rDer p 2–treated PBMCs. **B**, The proportion of FoxP3^+^ and FoxP3^−^ subsets within the CD25^+^LAG-3^+^CD4^+^ T cells. **C**, The percentage of CD49b^+^LAG-3^+^CD4^+^ T cells (Tr1). **D**, The percentage of PD-1^+^LAG-3^+^CD4^+^ T cells (n = 15). All results are shown as mean ± SD. *NS*, Not significant (Wilcoxon matched-pairs test). ∗*P* < .05; ∗∗*P* < .01.

Treg cells, especially the FoxP3^+^ and CD49b^+^LAG-3^+^ (Tr1) subsets, are known to play a role in the development of tolerance in AIT.^12^^,^^15^, ^16^, ^17^ We therefore assessed whether sia-rDer p 2 could expand Treg cells. Given the vast phenotypic heterogeneity of human Treg cells, we analyzed only the following subsets: CD4^+^CD25^+^FoxP3^+^, CD25^+^LAG-3^+^, Tr1, PD-1^+^, and PD-1^+^LAG-3^+^ Treg cells. For the gating strategy, see Fig E3 (in the Online Repository available at www.jaci-global.org). sia-rDer p 2 did not affect the frequencies of CD25^+^FoxP3^+^ Treg cells (Fig E3) nor the levels of PD-1 expression (Fig E3). However, compared with rDer p 2, sia-rDer p 2 expanded the frequencies of CD25^+^LAG-3^+^ (Fig 5, *A*), Tr1 (Fig 5, *C*), and PD-1^+^LAG-3^+^ (Fig 5, *D*) Treg cells. Interestingly, among the CD25^+^LAG-3^+^ lymphocytes, only the FoxP3^−^ subset was expanded in sia-rDer p 2–treated PBMCs but not the FoxP3^+^ subset (Fig 5, *B*). Collectively, these data indicate that sia-rDer p 2 but not native rDer p 2 increases the frequency of LAG-3^+^ CD4^+^ T cells.

### Treatment of PBMCs with sia-rDer p 2 before treatment with rDer p 2 downregulates T_H_2 cytokine production and CD4^+^ T-cell activation

**Fig 6:**
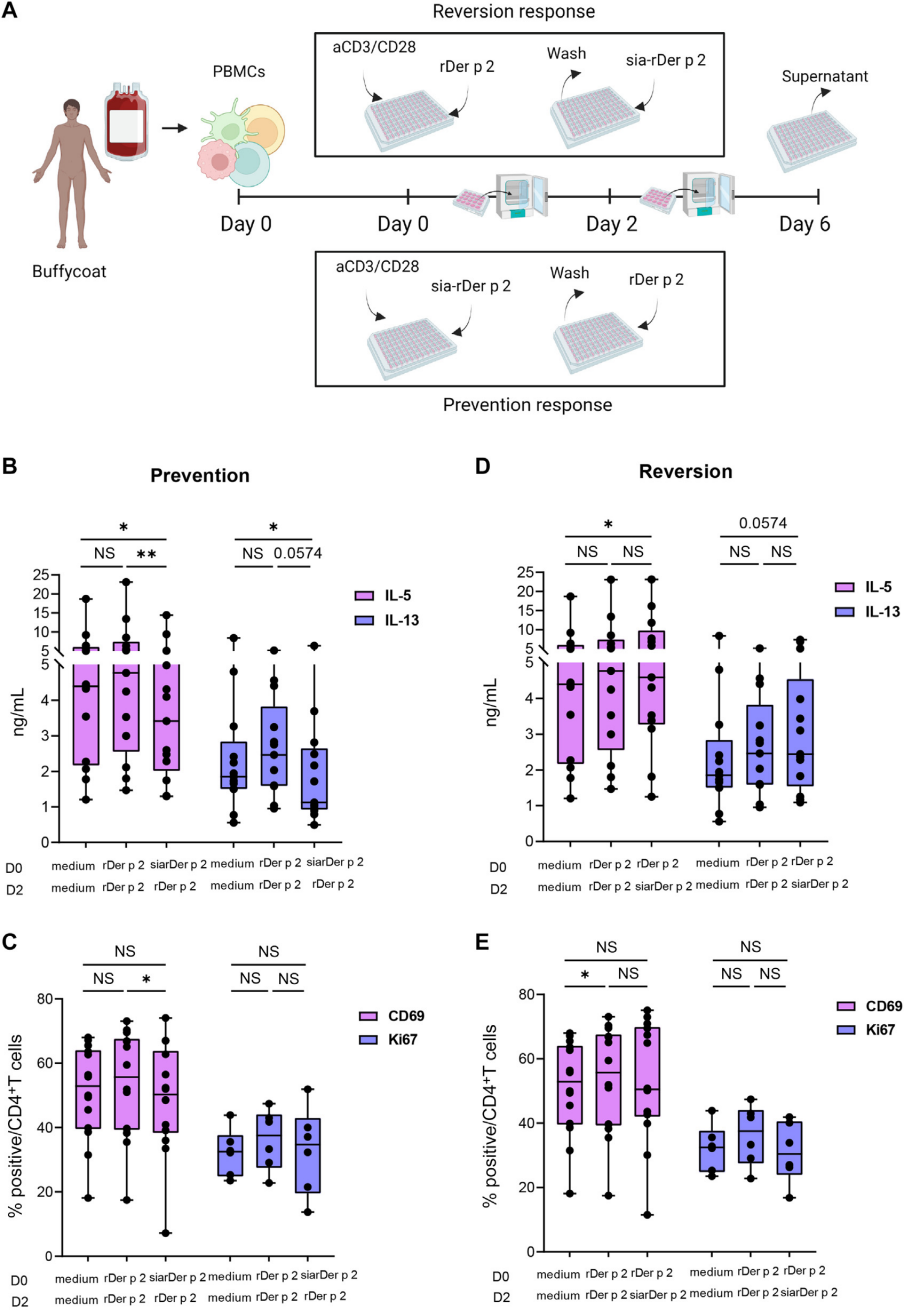
Prophylactic treatment of PBMCs with sia-rDer p 2 before challenge with rDer p 2 downregulates IL-5 and IL-13 production. **A**, A schematic representation of the *in vitro* experimental setup to investigate the effect of sia-rDer p 2 in a prophylactic (prevention response) and therapeutic (reversion response) setting. **B-E**, IL-5 (*pink boxes*) and IL-13 (*purple boxes*) levels (n = 11) were measured in day 6 PBMC supernatant by ELISA in the prevention response (Fig 6, *B*) and the reversion response (Fig 6, *D*). The percentage of CD69^+^ (*pink boxes*) (n = 11) and Ki67^+^ (*purple boxes*) (n = 5), CD4^+^ T cells in day 6 PBMCs in the prevention response (Fig 6, *C*) and the reversion response (Fig 6, *E*). All results are shown as mean ± SD. *NS*, Not significant (Wilcoxon matched-pairs test). ∗*P* < .05; ∗∗*P* < .01.

Following our observation that sia-rDer p 2 suppresses the expression of CD69 (Fig 2, *C*) and the production of IL-5 (Fig 2, *C*, and Fig 3, *C*), we investigated its capacity to either prevent or revert CD4^+^ T-cell activation in rDer p 2–treated PBMCs. To do this, anti–CD3-/CD28-activated PBMCs were exposed to sia-rDer p 2 either 48 hours before (prevention of response) or after (reversal of response) coincubation with rDer p 2 (Fig 6, *A*). We observed that treating PBMCs with sia-rDer p 2 before treating with rDer p 2 resulted in the downregulation of both IL-5 and IL-13 (Fig 6, *B*). However, the percentages of both CD69^+^CD4^+^ and Ki67^+^CD4^+^ T cells (Fig 6, *C*) were unaltered. In contrast, treating PBMCs with sia-rDer p 2 after pretreating them with rDer p 2 resulted in an increase in IL-5 and IL-13 (Fig 6, *D*). Nonetheless, the proportions of CD69^+^CD4^+^ and Ki67^+^CD4^+^ T cells appeared to be reduced (Fig 6, *E*). Together, these data demonstrate that sia-rDer p 2 can prevent the activation of T_H_2 cytokine responses on challenge with rDer p 2.

## Discussion

This study aimed to investigate the immunomodulatory capacity of sialic acid–modified recombinant Der p 2 on the T_H_2 arm of CD4^+^ T cells in human PBMCs from nonallergic individuals *ex vivo*. We also evaluated whether sia-rDer p 2 could induce the expansion of Treg cells. We noted that sia-rDer p 2 moderately suppressed the activation of CD4^+^ T cells, evidenced by a reduction in the expression of CD69 in both T_H_1 and T_H_2 cells and downregulated the production of T_H_2 cytokines IL-5 and IL-13 but not the T_H_1 cytokine IFN-γ or the T_H_17 cytokine IL-17. We also showed that sia-rDer p 2 binds to Siglec-9 present on monocytes, DCs, and B cells, and that these cells may be involved in the suppression of CD4^+^ T-cell activation and in the diminished production of T_H_2 cytokines.

The Der p 2 protein has gained attention as an alternative option to prevent and treat HDM allergy besides crude HDM extracts.^3^^,^^18^, ^19^, ^20^ This is because there is still ongoing debates about properly standardizing the components of the crude HDM extracts that are currently used in clinics,^21^ and the risk of unwanted side effects is still quite high.^22^ Moreover, 79.2% of people with asthma have high IgE titers specific for Der p 2,^23^ and Der p 2 itself is a strong risk factor for the development of asthma.^10^ Moreover, Der p 2 is a good model antigen for investigating the effects of sialic acid–protein modification on immune responses because it contains 12 free lysine residues on which sialic acid molecules could be conjugated. In sialic acid–Siglec interactions, the use of multivalent ligands is necessary to achieve sufficient avidity to stably bind Siglecs and subsequently transduce strong immunomodulatory signals.^24^ We hypothesized using Der p 2 decorated with sialic acids instead of native Der p 2 would prevent the initial spike in T_H_2 responses^25^ observed during HDM AIT and potentially decrease the time needed to develop full tolerance because of the induction of immunomodulatory responses mediated by the sialic acid–Siglec axis.

We observed that when PBMCs from nonallergic individuals were treated with rDer p 2, they produced more IL-4 and IL-13, features that are typical of an allergic phenotype. Indeed, nonatopic individuals have been reported to possess allergen-specific cells,^26^^,^^27^ although the response to the allergen is of a lower intensity compared with those of atopic individuals.^28^ This could explain the observed increase in IL-4 and IL-13. Notably, however, the production of IL-4 and IL-13 from sia-rDer p 2–treated PBMCs from the same nonallergic individuals was unaltered, whereas the production of IL-5 was downregulated. This indicates that sia-rDer p 2 may be safer than native Der p 2 because it does not enhance T_H_2 cytokine production. In addition, sia-rDer p 2 did not affect the production of both IL-17 and IFN-γ, which can enhance the severity of allergic diseases and asthma.^29^^,^^30^ In an *in vitro* prophylactic setting, where we treated PBMCs with sia-rDer p 2 before treating them with rDer p 2, sia-rDer p 2 downregulated the production of IL-5 and IL-13. Collectively, these data show that sia-rDer p 2 inhibits T_H_2 cytokine production, especially IL-5, and may therefore be a good candidate for Der p 2–specific AIT or be used for prophylactic vaccination against Der p 2–HDM allergy.

When tested in an *ex vivo* therapeutic setting (PBMCs were first treated with rDer p 2 and then with sia-rDer p 2), sia-rDer p 2 did not produce the suppressive effects described earlier. We hypothesize that the activation signal produced by a combination of anti-CD3/CD28 and rDer p 2 was too strong to be overcome by sia-rDer p 2. The fact that lowering the amounts of anti-CD3/CD28 did not result in a different outcome (data not shown) might indicate that the changes provoked in the PBMCs after activation with anti-CD3/CD28 cannot be reversed by the immunosuppressive signals generated by sialic acids. Indeed, anti-CD3–mediated T-cell activation can be overridden only when the suppressive compound is administered simultaneously with anti-CD3.^31^ Notably, the fact that sia-Der p 2 modulated only T_H_2 cytokines, and not IFN-γ or IL-17, as opposed to previous reports with ovalbumin as antigen,^7^ indicates that the activity of sia-Der p 2 may be driven by APCs. Moreover, the involvement of APCs is also substantiated by our finding that sia-rDer p 2 does not affect T_H_2 cytokine secretion when incubated with pure CD4^+^ T cells.

Beyond the T_H_2 cytokine response, we measured the effect of sia-rDer p 2 on the activation of CD4^+^ T cells by the expression of CD25 and CD69. CD25 is widely recognized as the primary marker for cellular activation,^32^ whereas a prolonged increase in the expression of CD69 is associated with allergic inflammation.^33^ Our findings show that sia-Der p 2 markedly suppressed the expression of CD69. The effects of sia-rDer p 2 on CD69 were similar in both T_H_1 and T_H_2 cells. Shinoda et al^34^ reported that CD69-deficient CD4^+^ T cells cannot help B cells to produce high-affinity antibodies and fail to mediate the generation of long-lived plasma cells. Therefore, immunotherapy with sia-rDer p 2 would indirectly inhibit the generation of long-lived allergic plasma cells.

Another important mechanism that has been proposed to be involved in successful AIT is the induction of Treg cells.^12^^,^^22^ The 2 most commonly described subsets of Treg cells that play a key role in allergen tolerance are FoxP3^+^ Treg cells^35^^,^^36^ and IL-10^+^ Tr1 cells.^16^^,^^37^ We showed that, compared with rDer p 2, treatment of PBMCs with sia-rDer p 2 did not affect the frequency of FoxP3^+^ Treg cells but expanded the frequency of Tr1 cells. Of note, Tr1 cells, having strong suppressive activity, are expanded after AIT, and they correlate with a decrease in clinical scores.^16^ Because LAG-3 is increasingly implicated in the downregulation of T-cell responses,^38^^,^^39^ we measured the frequency of LAG-3^+^ Treg-cell subsets. We observed that the frequencies of CD25^+^LAG-3^+^ Treg cells, notably the FoxP3^−^ subset and PD-1^+^LAG-3^+^, were higher in the sia-rDer p 2 than in the rDer p 2 condition. Dawicki et al^40^ have reported that CD25^+^LAG-3^+^FoxP3^−^ Treg cells can contribute to tolerance induction. These data suggest that AIT with sia-rDer p 2 instead of Der p 2 might maintain the abundance of necessary Treg-cell populations, which may accelerate the development of tolerance to Der p 2.

Overall, we have demonstrated that α2-3 sialic acid–modified rDer p 2 is able to suppress the activation of both T_H_1 and T_H_2 cells and downregulate the production of T_H_2 cytokines IL-5 and IL-13 in PBMCs from nonallergic individuals. Moreover, sia-rDer p 2, unlike rDer p 2, does not enhance the activation and proliferation of CD4^+^ T cells, does not alter the Treg/T_H_2 cytokine balance, and does not alter the frequency of Treg cells. All these findings illustrate that the use of sia-rDer p 2 instead of rDer p 2 for Der p 2–specific AIT would potentially be more beneficial especially in the early phase of AIT, which is often characterized by a heightened T_H_2 response and corresponding allergic side effects, although this needs to be confirmed using cells from subjects with established HDM allergy. Further studies, especially in cells from HDM-allergic donors, are needed to broaden the knowledge of the present findings and to confirm their possible use in routine clinical practice.

**Clinical implications**

Our findings provide evidence that can support research into the development of a safe prophylactic compound against HDM allergy, which may accelerate the development of necessary regulatory responses.

## Disclosure statement

This study was funded by 10.13039/100010677HEALTH HOLLAND (HH LSHM19073) and DC4U Technologies.

Disclosure of potential conflict of interest: E. R. J. Li and Y. van Kooyk are involved in DC4U Technologies, which develops glycan-based technologies that enable steering the human immune response. R. van Ree receives consultancy fees from HAL Allergy BV, Citeq BV, Angany, Inc, Reacta Healthcare Ltd, AB Enzymes, Mission MightyMe, and The Protein Brewery; receives speaker fees from HAL Allergy BV, ALK, and Thermo Fisher Scientific; and possesses stock options at Angany, Inc. The rest of the authors declare that they have no relevant conflicts of interest.
