# Supplementary material for: Sialic acid–modified der p 2 allergen exerts immunomodulatory effects on human PBMCs
Source: J Allergy Clin Immunol Glob. 2023 Nov 20;3(1):100193. doi: 10.1016/j.jacig.2023.100193 (PMC10770723; doi:10.1016/j.jacig.2023.100193)
Supplement: Supplementary data [file mmc1.docx]

**ONLINE REPOSITORY**

**Sialic acid-modified Der p 2 allergen exerts immunomodulatory effects in human PBMC**

Brigitte-Carole Keumatio Doungtsop MSc^1,2^, Eleonora Nardini MSc ^1,2^, Hakan Kalay BSc^1,2^, Serge A. Versteeg MSc^2^, Joyce Lübbers PhD^1^, Gaby van Barneveld MSc^1^, Eveline RJ Li PhD^1,2,4^, Sandra J. van Vliet PhD^1,2^, Ronald van Ree PhD^2,3^, Esther C. de Jong PhD^2,3^, Yvette van Kooyk PhD^1,2,4*^

*^1^Amsterdam UMC location Vrije Universiteit Amsterdam, Department of Molecular Cell Biology and Immunology, De Boelelaan 1117, Amsterdam, the Netherlands*

*^2^Amsterdam institute for Infection and Immunity, Inflammatory diseases, Amsterdam, the Netherlands*

*^3^ Amsterdam UMC location Amsterdam Medical Center, Department of Experimental Immunology, Meibergdreef 9, 1105 AZ Amsterdam, Amsterdam, the Netherlands.*

*^4^DC4U technologies, Abcoude, Netherlands.*

*Corresponding author: Yvette van Kooyk, PhD, ^1^Amsterdam UMC location Vrije Universiteit Amsterdam, Department of Molecular Cell Biology and Immunology, De Boelelaan 1117, Amsterdam, the Netherlands, ^2^Amsterdam institute for Infection and Immunity, Inflammatory diseases, Amsterdam, the Netherlands, ^4^DC4U technologies, Abcoude, Netherlands*.* E-mail: [y.vankooyk@amsterdamumc.nl](mailto:y.vankooyk@amsterdamumc.nl)

**Materials and Methods**

**Modification of recombinant Der p 2 with Sialic acids**

To obtain sialic-acid conjugated rDer p 2 (Sia-rDer p 2), maleimide-activated 3′- sialyl-N-acetyllactosamine (SLN302; Neu5Acα2,3Galβ1,4Glc, DEXTRA Labs) were conjugated to thio-activated rDer p 2 (Amsterdam University Medical Center (AmsUMC)) and through a thiol-ene reaction. The glycans were activated with the bifunctional cross linker 4-N-maleimidophenyl butyric acid hydrazide (MPBH) (Pierce) and rDer p 2 was activated with the linker N-succinimidyl S-acetylthioacetate (SATA) (Pierce). The hydrazide moiety of MPBH was covalently linked to the reducing end of the carbohydrate via reductive amination at a 3:1 molar ratio, rDer p 2 was reacted with SATA via the amino groups on its surface at a 6:1 molar ratio, and the final reaction of rDer p 2-SATA with the derivatized carbohydrate was performed at a molar ratio of 1:10. Briefly, SATA, dissolved in DMSO, was added to a filtered solution of rDer p 2 in phosphate buffer (pH 8.2; filtered to 200 nm). After vigorous stirring, the resulting rDer p 2-SATA^Ac^ solution was purified using a PD10 column (GE Healthcare) and diluted with 10% vol/vol of 0.5 M NH2OH·HCl solution (pH 7.2). After another 40 min of vigorous stirring and a second purification using a disposable size exclusion cartridge (PD10 column; GE Healthcare Life Sciences), rDer p 2-SATA^SH^ was ready for coupling with the MPBH-glycans. A mixture of MPBH (3 eq), 3′-sialyl-N-acetyllactosamine (or 6′-sialyl-N-acetyllactosamine) (1 eq), and picoline borane complex (10 eq; Sigma-Aldrich) dissolved in DMSO/AcOH (8:2) was incubated for 2 h at 65 °C, cooled to room temperature (RT), 1.4 mL of ice-cold isopropanol (anhydrous; Sigma-Aldrich) was added, and then incubated at –20 °C for 1 h. Subsequently, the precipitated MPBH– carbohydrates were pelleted, washed twice with cold isopropanol, and dissolved in 50 μL of PBS. The derivatization was confirmed by electrospray ionization mass spectrometry. The obtained MPBH–3′ sialyl-N-acetyllactosamine was used immediately for coupling to rDer p 2-SATA^SH^. Conjugation of rDer p 2-SATA^SH^ to the activated glycans was performed o/n at 4 °C, and the neo-glycoconjugates were purified by size exclusion chromatography. The concentration of rDer p 2 was determined using the bicinchoninic acid assay (Pierce). The presence of α2-3-linked sialic acids on rDer p 2 was confirmed by ELISA (Fig. S1). Briefly, NUNC maxisorb plates (Thermo Fisher Scientific, USA) were coated overnight at room temperature (RT) with 10 µg/ml of either sia-rDer p 2 or non-conjugated rDer p 2 (r*Der p 2*). A polyacrylamide polymer containing α2-3 sialic acid (PAA-α2-3) (GlycoNZ, New Zealand) was used a positive control. After overnight incubation, the wells were blocked with carbo-free blocking buffer (CFBB) (Vector Laboratories, USA) for 1 hour at RT. Plates were then incubated with biotinylated MAL-I and peroxidase-labeled streptavidin (Sigma-Aldrich) was use to quantify binding.

**Amino Acid Sequence of Der p 2**

MMYKILCLSLLVAAVARDQVDVKDCANHEIKKVLVPGCHGSEPCIIHRGKPFQLEAVFEANQNTKTAKIEIKASIDGLEVDVPGIDPNACHYMKCPLVKGQQYDIKYTWNVPKIAPKSENVVVTVKVMGDDGVLACAIATHAKIRD

**Supplementary Figures (FigS)**


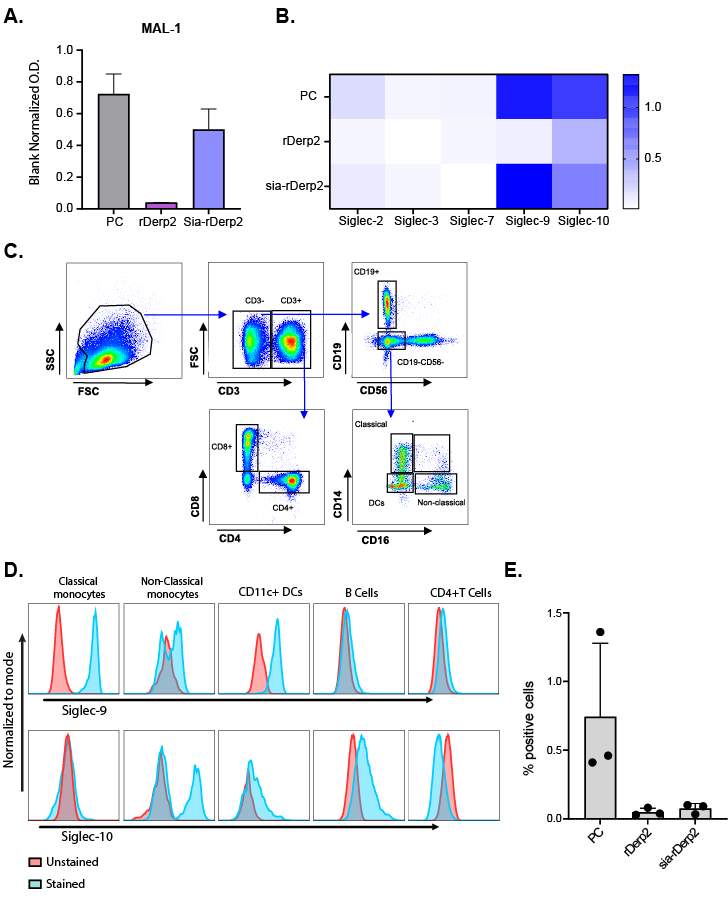


**Fig E1.** Binding of α2-3 sialic acid-rDer p 2 to Siglec-9 and -10. **A,** Binding of polyacrylamide (PAA)-α2-3 sialic acid (PC), native rDer p 2 (rDer p 2), and α2-3 sialic acid-conjugated rDer p 2 (sia-rDer p 2) to *Maackia amurensis* lectin I (MAL-1). **B**, Binding of PC, rDer p 2, and sia-rDer p 2 to different Siglec-Fc chimeras. **C,** Gating strategy used to identify monocytes, dendritic cells (DCs), B cells and CD4+T cells in PBMCs. **D,** Representative histograms showing the expression of Siglec-9 and -10 on different immune cell populations within PBMCs. **E,** Binding of PC, rDer p 2, and sia-rDer p 2 to CD4+T cells.


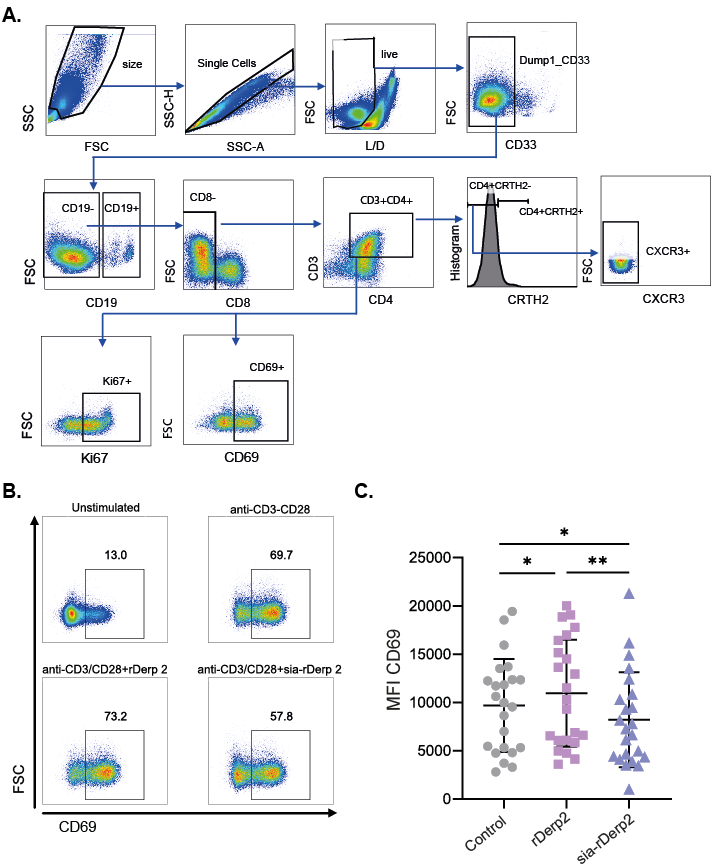


**Fig E2.** Activation and proliferation of α2-3 sialic acid-rDer p 2 and rDer p 2-treated CD4+T cells. **A,** Gating strategy used to identify CRTH2+ (Th2), CXCR3+ (Th1), CD69+ and Ki67+ CD4+T cells in PBMCs left unactivated or activated with anti-CD3/CD28 antibodies and co-incubated with either RPMI medium (control), or rDer p 2, or sia-rDer p 2 and cultured for 6 days. **B,** Representative dot plot showing the percentage of CD69+CD4+T cells in PBMCs treated with medium (control), rDer p 2 or sia-rDer p 2. **C,** The mean fluorescent intensity (MFI) of CD69 on CD4+T cells. All results are shown as mean ± SD. * p <0.05; ** p< 0.01; Wilcoxon matched-pairs test.


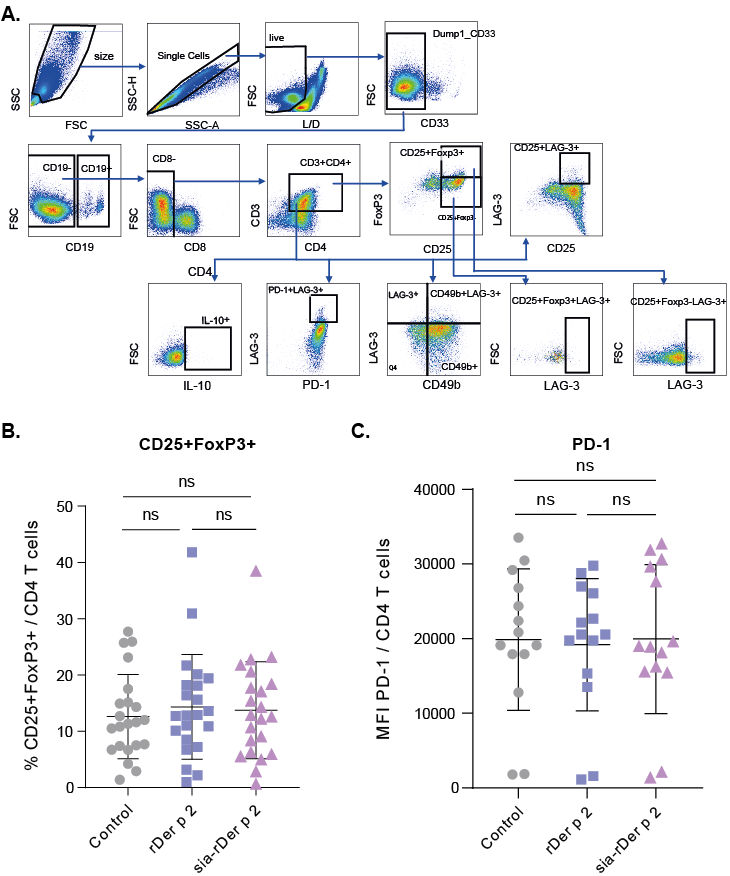


**Fig E3.** Different Treg populations in PBMCs treated with rDer p 2 or sia-rDer p 2. Non-allergic PBMCs were left unactivated or activated with anti-CD3/CD28 antibodies, co-incubated with either RPMI medium (control), or rDer p 2, or sia-rDer p 2 and cultured for 6 days. **A,** Gating strategy used to quantify CD19+IL-10+, CD4+IL-10+, PD-1+LAG-3+, CD49b+LAG-3+, FoxP3-/FoxP3+ CD25+LAG-3+ lymphocytes. **B,** The percentage of CD25+Foxp3+ CD4+T cells. **C,** The MFI of PD-1 on CD4+T cells. Results are shown as mean ± SD. ns, not significant, Wilcoxon matched-pairs test.
